# Proteomic Insights into Antibiotic Resistance and Biofilm Adaptation of *Staphylococcus epidermidis* to Subinhibitory Levofloxacin, Rifampicin, and Combination Treatment

**DOI:** 10.3390/pathogens15101008

**Published:** 2026-09-24

**Authors:** Kidon Sung, Miseon Park, Jungwhan Chon, Ohgew Kweon, Saeed Khan, Steven L. Foley

**Affiliations:** 1 Division of Microbiology, National Center for Toxicological Research, U.S. Food and Drug Administration, Jefferson, AR 72079, USA; miseon.park@fda.hhs.gov (M.P.); oh-gew.kweon@fda.hhs.gov (O.K.); saeed.khan@fda.hhs.gov (S.K.); steven.foley@fda.hhs.gov (S.L.F.); 2 Department of Food Science and Biotechnology, Kangwon National University, Chuncheon 24341, Republic of Korea; alvarmar@naver.com

**Keywords:** biofilm, levofloxacin, rifampicin, proteome, *Staphylococcus epidermidis*

## Abstract

*Staphylococcus epidermidis* biofilms are inherently tolerant to antimicrobial treatment, contributing to the persistence and recurrence of biofilm-associated infections. In this study, we examined the phenotypic and proteomic responses of *S. epidermidis* ATCC 35984 biofilms following exposure to subinhibitory concentrations (1/8× MIC) of levofloxacin, rifampicin, and their combination. Phenotypic analyses revealed clear differences among the treatments. Rifampicin significantly reduced the number of viable biofilm-associated cells, whereas levofloxacin alone had little effect. The combination of rifampicin and levofloxacin produced the most pronounced antibiofilm activity, significantly reducing both biofilm biomass and viability. These findings were supported by confocal laser scanning microscopy, which showed the greatest loss of viable cells and biofilm integrity in the combination-treated biofilms. Label-free quantitative proteomic analysis demonstrated distinct treatment-specific adaptive responses. Rifampicin, either alone or in combination with levofloxacin, triggered broadly increased abundance of proteins involved in translation, energy production, amino acid and nucleotide metabolism, ATP-binding cassette transport, and quorum sensing. In contrast, levofloxacin predominantly suppressed proteins associated with central metabolism, transcription, translation, and biosynthetic pathways. Hierarchical clustering further showed that the proteomic profile of the combination treatment closely resembled that of rifampicin alone, suggesting that rifampicin was the dominant driver of proteomic remodeling. Expression pattern network analysis identified a conserved stress-response signature comprising Fhs, YdaG, PerR, and YcsE across all treatment conditions. Notably, the multidrug efflux pumps NorA and NorC showed decreased protein abundance across all treatment conditions, indicating a potential reduction in efflux-mediated resistance under sub-MIC antibiotic exposure. Taken together, these findings demonstrate that rifampicin retains substantial antibiofilm activity at subinhibitory concentrations and elicits extensive metabolic adaptation, whereas levofloxacin primarily induces a metabolically repressed state. The enhanced activity of the rifampicin–levofloxacin combination appears to arise from complementary effects on biofilm viability and cellular physiology. This study provides new insights into the adaptive responses of *S. epidermidis* biofilms to sublethal antibiotic stress and highlights the potential of rifampicin-based combination therapies for the management of biofilm-associated infections.

## 1. Introduction

Infections associated with implanted medical devices pose a serious clinical challenge, due to the formation of bacterial biofilms that complicate treatment [1]. Devices such as central venous catheters, artificial joints, pacemakers, and heart valves are particularly susceptible to biofilm colonization. Once established, these infections are often persistent and difficult to eliminate. *Staphylococcus epidermidis* is one of the most frequently isolated pathogens and is known to be a major cause of biofilm-related infections on medical devices [1].

The initiation of these infections typically involves initial attachment of bacteria to the surface of the implanted material, a process mediated by specific bacterial surface proteins [2]. Because *S. epidermidis* inhabits protected skin niches such as sebaceous glands and sweat ducts, it can persist despite standard preoperative skin disinfection and antimicrobial prophylaxis measures [3]. Even a small number of these bacteria introduced during surgery can attach to the surface of the device, multiply, and cause infection. In catheter-related infections, bacteria can travel along the surface of the device and disseminate into the bloodstream [4].

A major reason these infections persist is the formation of biofilms, in which bacteria become encased in a self-produced extracellular matrix [5]. In *Staphylococcus* species, the biofilm matrix often contains poly-N-acetylglucosamine, which helps maintain structural integrity and protects the cells from external stresses [6]. Within biofilms, *S. epidermidis* exhibits reduced metabolic activity and a sessile lifestyle, which results in significant phenotypic heterogeneity [6]. These changes make the bacteria much more resistant to antibiotics and immune system responses than planktonic bacteria. For example, *Pseudomonas aeruginosa* biofilms formed on urinary catheters have been shown to be up to 2500 times more resistant to tobramycin than planktonic cells [7]. Biofilm-associated infections tend to become chronic and are estimated to account for at least 65% of persistent bacterial infections [8].

Despite advances in managing biofilm-related infections, the impact of sub-MIC antibiotic exposure on established biofilms is still not well understood. At concentrations below the minimum inhibitory concentration (sub-MIC), antibiotics can affect bacterial behavior, including alterations in surface adhesion and biofilm development [9]. For example, sub-MIC levels of rifampicin have been reported to promote biofilm formation in *S. epidermidis* [10], whereas azithromycin and clarithromycin have been shown to inhibit biofilm formation in *P. aeruginosa* [11,12]. Despite these observations, little is known about how low-dose antibiotic exposure alters global protein expression and virulence properties of coagulase-negative staphylococci, including *S. epidermidis*. Rifampicin and levofloxacin were selected because they act through different but complementary mechanisms. Rifampicin inhibits bacterial RNA polymerase and blocks transcription, whereas levofloxacin targets DNA gyrase and topoisomerase IV, thereby interfering with DNA replication. Rifampicin-based combinations are also used in the treatment of staphylococcal device-associated infections, where combining antibiotics with different targets may help limit the emergence of resistance and improve activity against biofilms [13]. To characterize both functional and molecular responses, we evaluated the effects of subinhibitory levofloxacin, rifampicin, and their combination on the viability, biomass, and proteome of preformed *S. epidermidis* ATCC 35984 biofilms.

## 2. Materials and Methods

### 2.1. Bacterial Strain, Minimum Inhibitory Concentration (MIC) Determination, and Biofilm Culture Conditions

*S. epidermidis* ATCC 35984 was inoculated into tryptic soy broth (TSB) (Thermo Fisher Scientific, Waltham, MA, USA) and cultured at 37 °C for 18 h with continuous shaking at 200 rpm in a refrigerated incubator shaker (Eppendorf, Hauppauge, NY, USA). The culture was diluted 1:500 in TSB (Thermo Fisher Scientific) supplemented with 0.25% glucose (TSBg) and grown to the logarithmic phase. Bacterial cells were harvested by centrifugation, the supernatant was discarded, and the cell pellet was washed with phosphate-buffered saline (PBS; Thermo Fisher Scientific).

The minimum inhibitory concentrations (MICs) of levofloxacin and rifampicin against *S. epidermidis* were determined using the broth microdilution method [14]. Two-fold serial dilutions of each antibiotic were prepared using cation-adjusted Mueller-Hinton II Broth (Thermo Fisher Scientific). A standardized *S. epidermidis* inoculum corresponding to a 0.5 McFarland standard was added to each well of a 96-well microplate (Thermo Fisher Scientific). The inoculated microplates were sealed with parafilm (Thermo Fisher Scientific) and incubated at 37 °C with continuous linear shaking in an Agilent BioTek Synergy HTX multi-mode plate reader (Agilent Technologies, Santa Clara, CA, USA). Bacterial growth was assessed for 24 h by measuring the absorbance at 600 nm (OD600). The MIC was defined as the lowest concentration of each antibiotic that completely inhibited bacterial growth based on spectrophotometric data. All MIC measurements were performed in biological triplicate.

For biofilm formation, the bacterial suspensions were adjusted to a turbidity corresponding to the 0.5 McFarland standard. The suspensions were inoculated into 96-well plates and incubated at 37 °C for 24 h with shaking at 130 rpm in the same shaker. After biofilm formation, planktonic cells were removed. The biofilms were exposed to sub-MICs of antimicrobial agents, including levofloxacin (0.031 µg/mL, 1/8× MIC), rifampicin (0.0025 µg/mL, 1/8× MIC), or a combination of these two antibiotics. Control wells were not treated with antimicrobial agents. All treatments were performed at 37 °C for 24 h with gentle shaking. After treatment, planktonic cells were removed and biofilm cells were gently washed with PBS. Biofilms were then collected and transferred to microcentrifuge tubes. Collected biofilm cells were centrifuged, washed with PBS, and stored at −80 °C for subsequent protein extraction.

### 2.2. Quantification of Biofilm Biomass by Crystal Violet Assay

Biofilm formation of *S. epidermidis* ATCC 35984 was quantified using a crystal violet staining assay [15]. Briefly, after biofilm formation and treatment, as described above, planktonic cells were removed and biofilms were washed with PBS. The attached biofilms were stained with 125 µL of 0.1% 0.22 µm-filtered crystal violet solution (MilliporeSigma, Burlington, MA, USA) for 15 min at room temperature. After washing to remove excess stain and air-drying, the bound crystal violet was solubilized in 125 µL of 30% acetic acid solution (MilliporeSigma) for 15 min. The solubilized crystal violet was quantified by absorbance at 600 nm using the plate reader (Agilent Technologies). Data were visualized using box plots generated in OriginPro (OriginLab Corporation, Northampton, MA, USA). Boxes represent the interquartile range (IQR; 25th–75th percentiles), with the median shown as a horizontal line and the mean indicated by an open square. Whiskers extend to values within 1.5 × IQR, and points outside this range are plotted as outliers. Statistical analysis was performed using one-way ANOVA with Tukey’s Honest Significant Difference (HSD) post hoc test [16].

### 2.3. Quantification of Viable Cells in Biofilms

Viable cells in *S. epidermidis* ATCC 35984 biofilms were quantified by enumerating colony-forming units (CFUs) as previously described [17]. Briefly, after biofilm formation and antibiotic treatment as described above, planktonic cells were removed and biofilms were gently washed with PBS. The attached biofilms were collected using a sterile cotton swab moistened with PBS and transferred to a 15 mL centrifuge tube containing 2 mL of PBS. Biofilms were then detached by vortexing for 5 min, followed by sonication for 5 min at 40 kHz (Emerson, St. Louis, MO, USA), and finally vortexing for 1 min. Serial dilutions were prepared in PBS and plated on tryptic soy agar (TSA; Thermo Fisher Scientific) supplemented with 5% defibrinated sheep blood. Plates were incubated at 37 °C for 18–24 h, and CFUs were enumerated using a ProtoCOL 3 automated colony counter (Synbiosis, Frederick, MD, USA). Box plot graphing and statistical analyses were performed as described above.

### 2.4. Confocal Laser Scanning Microscopy (CLSM)

Biofilms were prepared under the conditions described above. They were washed with PBS and stained for 25 min at room temperature with a FilmTracer LIVE/DEAD Biofilm Viability Kit (Molecular Probes, Eugene, OR, USA). Following staining with SYTO 9 and propidium iodide, the biofilms were gently rinsed with filter-sterilized water. CLSM images were acquired using an Agilent Biotek Cytation 10 equipped with Gen5 Image Prime software (version 3.16; Agilent Technologies). Imaging was carried out using manual mode with an LED intensity of 10, an integration time of 5 milliseconds, and a camera gain of 32 with a 20× Plan Fluor (PL) objective with a 60 µm confocal disk. Fluorescence signals were detected using GFP (excitation 469 nm, emission 525 nm) and TRITC (excitation 543 nm, emission 593 nm) filter cubes.

### 2.5. Proteomic and Bioinformatic Analysis

Biofilm cells were lysed by combining mechanical bead-beating and chemical disruption, followed by heat denaturation at boiling temperatures [18]. Protein samples were reconstituted in lysis buffer containing SDS, Tris-HCl, Tris(2-carboxyethyl)phosphine, and 2-chloroacetamide [19]. All chemical reagents were purchased from MilliporeSigma. Protein digestion was performed using a single-pot solid-phase-enhanced sample preparation method [9]. The digested peptides were analyzed by ultra-high performance liquid chromatography-tandem mass spectrometry (UHPLC-MS/MS; Thermo Fisher Scientific) [20].

For the proteomic analysis, one biological sample was analyzed for each condition. Because biological replicates were not included in the proteomic analysis, statistical comparisons based on replicate measurements could not be performed. The proteomic analysis was used to examine overall changes in protein abundance across the different treatment conditions.

Liquid chromatography (LC) was performed using an UHPLC system coupled to a quadrupole-orbitrap mass spectrometer. The mass spectrometer (MS) was operated in data-dependent acquisition (DDA) mode, selecting the top 12 precursor ions for fragmentation in the mass scan range of 350–1400 m/z. Collision energy, automatic gain control, and maximum fill time of MS1 and MS2 were optimized for peptide fragmentation.

Data processing and database searching followed established methods [21]. MS1-based isotope features were detected using OpenMS software version 3.5.0, and protein abundance was quantified by calculating peptide peak areas [22]. Protein identifications were retained only when supported by at least one unique peptide and an E-value below 0.0001. Proteins with a fold change of ≥2.0 were classified as showing increased abundance, while proteins with a fold change of ≤0.5 were classified as showing decreased abundance, compared to the control and treatment groups. Because biological replicates were not available, the fold-change thresholds were used to describe treatment-related changes rather than to establish statistical significance.

Hierarchical clustering and heatmap visualization of the proteins identified were performed in Python (version 3.x) using the seaborn and matplotlib libraries. Protein expression values were normalized using a row-wise Z-score transformation. For each protein, the mean expression across the three antibiotic treatment conditions was subtracted from the individual expression values and then divided by the corresponding standard deviation. This approach standardized each protein to a mean of zero and a standard deviation of one, allowing relative expression patterns to be compared without the confounding influence of differences in absolute abundance. Proteins that showed no measurable variation across treatments were assigned a Z-score of zero.

The normalized data matrix was visualized with seaborn.clustermap using the *RdBu_r* diverging color palette, where red represents expression levels above the protein’s mean and blue represents levels below it. Hierarchical clustering was applied to both proteins (rows) and treatment conditions (columns) using Euclidean distance and average linkage. To highlight meaningful shifts in expression, the color scale was constrained to ±1.5 Z-score units. Protein clusters were then defined using the scipy.cluster.hierarchy.fcluster function, based on a predefined maximum number of clusters.

Shared and unique protein expression profiles across treatment groups was visualized using a Venn diagram generated with InteractiVenn [23]. Differentially expressed proteins were functionally classified using the Cluster of Orthologous Groups (COG) [24] and mapped to metabolic pathways using the Kyoto Encyclopedia of Genes and Genomes (KEGG) [25]. Temporal protein expression profiles across treatment conditions were clustered using expression pattern network (EPN) analysis implemented in a custom Python script [26].

## 3. Results

### 3.1. Impact of Sub-MIC Levofloxacin and Rifampicin on S. epidermidis Biofilm Biomass and Viability

The MICs of levofloxacin and rifampicin against *S. epidermidis* ATCC 35984 were determined to be 0.25 and 0.002 µg/mL, respectively (Figure 1 and Figure 2). Biofilm biomass analysis showed that combination treatment produced a statistically significant reduction compared to control (*p* < 0.05), whereas levofloxacin or rifampicin mono-treatment had no significant effect (Figure 3). Viable cell count analysis revealed that rifampicin alone and the combination treatment significantly reduced viable biofilm cells relative to the control (*p* < 0.001), with the combination treatment showing the most pronounced effect (Figure 4).

CLSM analysis confirmed the quantitative biofilm findings following treatment with sub-MICs of levofloxacin, rifampicin, and their combination (Figure S1). The untreated control biofilm displayed a dense and homogeneously distributed structure with intense green fluorescence, indicating a predominance of viable cells within the biofilm matrix. Biofilms treated with levofloxacin showed only minimal structural and fluorescence changes compared with the control, suggesting that most cells remained viable, consistent with the limited effects observed in CFU assay.

In contrast, treatment with rifampicin led to a noticeable reduction in green fluorescence, indicating decreased cell viability and partial disruption of the biofilm structure. The combination of levofloxacin and rifampicin produced the greatest antibiofilm effect, characterized by reduced live biofilm cells. These visual observations were consistent with the quantitative findings, which demonstrated a significant reduction in viable biofilm-associated cells following combination treatment.

These findings indicate that rifampicin retains antibiofilm activity even at subinhibitory concentrations, and that this activity is further enhanced when combined with levofloxacin. While rifampicin mono-treatment substantially reduced cell counts, it did not have notable impact on the total biofilm biomass. The greater antibiofilm effect observed with the combination treatment likely reflects complementary mechanisms of action.

Previous studies have reported variable effects of sub-MIC rifampicin on staphylococcal biofilms. Schadow et al. reported that sub-MIC rifampicin mono-treatment strongly prevented the attachment of *S. epidermidis* ATCC 35984 and RP12 strains, but there were significant differences between the strains [10]. Ozturk et al. similarly found a general trend of biofilm suppression in *S. aureus* treated with sub-MIC rifampicin [27]. However, Park et al. and Lima-e-Silva et al. reported contrasting results, who observed increased biofilm formation in *S. aureus* after sub-MIC rifampicin exposure [28,29]. This suggests that the biofilm effect of rifampicin varies depending on the bacterial strain and environment. For levofloxacin, Jo and Ahn observed less than a 0.5 log CFU/mL reduction in *S. aureus* biofilms treated at sub-MIC [30], which is consistent with our observation that levofloxacin treatment had minimal effect at subinhibitory concentration. Collectively, these findings highlight the complex and context-dependent nature of biofilm responses to sublethal antibiotic exposure and support the enhanced antibiofilm efficacy of rifampicin when used in combination with levofloxacin.

### 3.2. Global Protein Expression Trends

We performed label-free quantitative proteomic analysis to examine the molecular basis of the observed phenotypic differences, which revealed distinct protein expression patterns across treatment groups. Rifampicin treatment, either alone or in combination with levofloxacin, induced increased abundance of 152 and 144 proteins, respectively (Figure 5, Table S1). In contrast, levofloxacin mono-treatment resulted in increased abundance of only 15 proteins.

Levofloxacin mono-treatment produced the most extensive decrease in protein abundance, with 166 proteins showing reduced abundance. Rifampicin mono-treatment resulted in decreased abundance of 25 proteins, while 76 proteins showed decreased abundance following combination treatment. Because biological replicates were not included in the proteomic analysis, the observed treatment-related changes were described using the predefined fold-change criteria and are not considered statistically significant.

The heatmap identified two major protein clusters with distinct expression profiles across the three antibiotic treatment conditions (Figure 6). Hierarchical clustering of the treatment groups showed that rifampicin and the levofloxacin–rifampicin combination clustered most closely together, whereas levofloxacin formed a separate branch. This clustering pattern suggests that rifampicin is the primary driver of the proteomic changes observed in the combination treatment.

Cluster 2 (*n* = 447), the largest protein cluster, was characterized by marked decrease in protein abundance in response to levofloxacin treatment, followed by moderate protein abundance under rifampicin and even stronger protein abundance in the combination-treated biofilms. These results suggest that proteins within this cluster are repressed by levofloxacin alone but are progressively induced in response to rifampicin, either as a single agent or in combination with levofloxacin. The stronger induction observed under combination treatment points to the activation of adaptive pathways involved in coping with antibiotic stress and promoting biofilm survival. COG analysis showed that Cluster 2 was enriched in proteins involved in translation, ribosomal structure, and biogenesis [J], amino acid transport and metabolism [E], energy production and conversion [C], nucleotide transport and metabolism [F], and carbohydrate transport and metabolism [G]. KEGG pathway analysis showed that the most prominently represented pathways in Cluster 2 were general metabolism (01100), cofactor biosynthesis (01240), secondary metabolite biosynthesis (01110), purine metabolism (00230), carbon metabolism (01200), and aminoacyl-tRNA biosynthesis (00970). The broad range of functions represented in this cluster is consistent with its large size and suggests that rifampicin, particularly when combined with levofloxacin, is associated with widespread increases in metabolic and biosynthetic activity, possibly reflecting a compensatory response to antibiotic-induced transcriptional stress.

In contrast, Cluster 3 (*n* = 240) exhibited minimal changes in expression following levofloxacin treatment, pronounced increase in protein abundance in response to rifampicin, and clear decrease in protein abundance under the levofloxacin–rifampicin combination. This expression pattern suggests that while rifampicin strongly induces this group of proteins, the addition of levofloxacin attenuates this response. The reduced expression of rifampicin-responsive proteins in the combination treatment may indicate that interactions between the two antibiotics modify the regulatory pathways involved in biofilm adaptation. COG analysis indicated that Cluster 3 was enriched in proteins associated with transcription [K], replication, recombination, and repair [L], posttranslational modification, protein turnover, and chaperones [O], as well as cell wall and membrane biogenesis [M]. KEGG pathway analysis further showed enrichment of the ABC transporter pathway (02010), quorum sensing (02024), one-carbon pool by folate (00670), and microbial metabolism in diverse environments (01120). This functional profile suggests that rifampicin is associated with increased abundance of proteins involved in regulation, stress responses, and cell wall-related processes, whereas the addition of levofloxacin appears to attenuate this response. This pattern may reflect an interaction between the two antibiotics that influence the cellular pathways involved in biofilm adaptation. Because Cluster 1 contained only 18 proteins, substantially fewer than Clusters 2 and 3, its contribution to the overall proteomic response is likely limited.

To further examine shared and treatment-specific proteomic responses, a Venn diagram was generated using the differentially expressed protein datasets (Figure 7). A total of 181, 177, and 220 proteins were differentially expressed in the levofloxacin, rifampicin, and combination treatment groups, respectively. Among these, 24 proteins were shared across all three conditions, suggesting a common proteomic response to sub-MIC antibiotic exposure in *S. epidermidis* biofilms. The levofloxacin mono-treatment group showed the highest number of uniquely expressed proteins (*n* = 117), followed by the combination treatment (89 proteins) and rifampicin mono-treatment (78 proteins). Only four proteins, YtrA (GntR family transcriptional regulator), DhaK (dihydroxyacetone kinase family protein), MnhA1 (Na^+^/H^+^ antiporter subunit A1), and Q5HMU5 (phage-associated protein), were common to both the levofloxacin and rifampicin mono-treatment groups.

### 3.3. COG-Based Functional Categorization

Differentially expressed proteins were classified using the Clusters of Orthologous Groups (COG) system to assess the functional distribution of the proteomic changes induced by sub-MIC antibiotic exposure. Proteins were classified into four major functional categories: cellular processes and signaling (D, cell cycle control, cell division, chromosome partitioning; V, defense mechanisms; T, signal transduction mechanisms; M, cell wall/membrane/envelope biogenesis; N, cell motility; U, intracellular trafficking, secretion, and vesicular transport; O, posttranslational modification, protein turnover, and chaperones), information storage and processing (J, translation, ribosomal structure, and biogenesis; K, transcription; L, replication, recombination, and repair), metabolism (C, energy production and conversion; G, carbohydrate transport and metabolism; E, amino acid transport and metabolism; F, nucleotide transport and metabolism; H, coenzyme transport and metabolism; I, lipid transport and metabolism; P, inorganic ion transport and metabolism; Q, secondary metabolite biosynthesis, transport, and catabolism), and poorly characterized functions (S, function unknown) [31].

Among the proteins showing increased abundance, rifampicin mono-treatment induced the broadest response across all functional categories (Figure 8A). The majority were linked to metabolism (68 proteins), followed by information storage and processing (33 proteins), cellular processes and signaling (26 proteins), and poorly characterized functions (19 proteins). A similar distribution was observed when rifampicin and levofloxacin were combined. In contrast, levofloxacin mono-treatment resulted in increased abundance of only a limited number of proteins.

Among the proteins showing decreased abundance, levofloxacin mono-treatment showed the most prominent effect (Figure 8B). A total of 75 metabolism-related proteins showed decreased abundance, together with 38 proteins involved in information storage and processing, 25 associated with cellular processes and signaling, and 23 classified as poorly characterized proteins. Combination treatment induced more modest decreases in protein abundance across all categories, with metabolism being most affected (25 proteins), followed by cellular processes and signaling (18 proteins), poorly characterized functions (17 proteins), and information storage and processing (14 proteins).

When examining specific COG categories, the most pronounced increase in protein abundance occurred in the translation, ribosome structure, and biogenesis categories [J], with 18 proteins showing increased abundance by rifampicin mono-treatment and 19 proteins upregulated by combination treatment (Figure 9A). Other categories that showed significant increase in protein abundance included nucleotide transport and metabolism [F] (18 and 12 proteins), amino acid transport and metabolism [E] (12 and 15 proteins), and energy production and conversion [C] (11 and 13 proteins). Levofloxacin mono-treatment showed increased abundance of only a few proteins in all categories.

Conversely, levofloxacin mono-treatment showed a notable suppression in several COG functional categories. The most significantly repressed groups were carbohydrate transport and metabolism [G] (18 proteins), nucleotide metabolism [F] (18 proteins), translation, ribosome structure, and biogenesis [J] (17 proteins), transcription [K] (14 proteins), energy production and conversion [C] (14 proteins), and amino acid metabolism [E] (12 proteins) (Figure 9B). Similar global metabolic suppression patterns were reported following fluoroquinolone exposure. Cirz et al. reported that ciprofloxacin treatment downregulated genes involved in nearly all aspects of *P. aeruginosa* metabolism [32]. This widespread metabolic suppression is consistent with the established mechanism of fluoroquinolones, which disrupt DNA replication and transcription [33].

### 3.4. KEGG Pathway Analysis

KEGG pathway analysis revealed that metabolic activity of *S. epidermidis* biofilms was markedly altered by sub-MIC antibiotic treatment. Rifampicin mono-treatment induced widespread and significant increases in protein abundance across numerous metabolic pathways. The most prominently affected category was general metabolism (KEGG: 01100), with 50 proteins showing increased abundance (Figure 10A, Tables S2 and S3). Additional significantly affected pathways included cofactor biosynthesis (01240; 24 proteins), secondary metabolite biosynthesis (01110; 22 proteins), one-carbon pool by folate (00670; 12 proteins), microbial metabolism in diverse environments (01120; 12 proteins), purine metabolism (00230; 11 proteins), and carbon metabolism (01200; 11 proteins). Similar metabolic activation has been reported in response to sublethal antimicrobial exposure. Rifampicin and clindamycin treatment significantly enhanced metabolic and biosynthetic pathways in *P. aeruginosa* biofilms, particularly those linked to secondary metabolites and amino acid biosynthesis [34]. In *S. epidermidis* biofilms, sublethal tigecycline exposure contributed to robust increases in the abundance of proteins associated with metabolic pathways, particularly those associated with cofactor and amino acid biosynthesis [9].

The combination of rifampicin and levofloxacin generated a metabolic response that closely resembled that of rifampicin alone, although the increase in protein abundance was somewhat less pronounced. This pattern suggests that rifampicin is the main driver of metabolic activation, while levofloxacin exerts only a modest influence on this response. By contrast, levofloxacin alone produced minimal metabolic overexpression.

Levofloxacin mono-treatment induced widespread decreases in protein abundance across KEGG-defined metabolic pathways. A total of 61 proteins showed decreased abundance in the general metabolism category (01100), with significant decreases also observed in secondary metabolite biosynthesis (01110; 24 proteins), microbial metabolism in diverse environments (01120; 21 proteins), amino acid biosynthesis (01230; 13 proteins), and carbon metabolism (01200; 12 proteins) (Figure 10B, Tables S2 and S3). Other pathways affected included purine metabolism (00230; 8 proteins) and cofactor biosynthesis (01240; 7 proteins).

Rifampicin mono-treatment exhibited minimal suppression confined to nucleotide and purine metabolism. Combination treatment showed a mixed proteomic response, with the increase in protein abundance associated with rifampicin partially compensated by the decrease observed with levofloxacin. This pattern likely reflects the combined effects of opposing mechanisms, indicating a more complex metabolic response under combination treatment.

While metabolic pathways dominated the overall response, important non-metabolic functional categories exhibited treatment-specific patterns. The ATP-binding cassette (ABC) transporter pathway (02010), a key membrane transport system, showed significantly increased abundance of OpuA, OpuC, and RbsD in rifampicin-treated and combination-treated biofilms. In contrast, levofloxacin alone did not significantly affect the expression of proteins within this pathway. This increase in protein abundance may reflect a stress response that enhances the transport of nutrients, signaling molecules, or toxic compounds. Previous studies have also linked increased ABC transporter expression to biofilm resilience following antibiotic exposure [35].

The aminoacyl-tRNA biosynthesis pathway (KEGG 00970), represented by ThrS, MetG, AspS, CysS, PheS, LysS, and AsnS, showed markedly increased protein abundance following rifampicin treatment, both alone and in combination with levofloxacin. The enhanced expression of these aminoacyl-tRNA synthetases likely reflects a compensatory response to rifampicin-mediated transcriptional inhibition.

The quorum sensing pathway (KEGG 02024), including SecA, SecE, FtsY, and Rot, showed increased abundance in response to rifampicin treatment, both alone and in combination with levofloxacin. Given its role in intercellular communication and biofilm regulation, this induction may reflect enhanced stress signaling and adaptive remodeling within the biofilm community.

### 3.5. EPN Cluster Analysis

Expression pattern network (EPN) clustering identified 15 distinct expression profiles in *S. epidermidis* biofilms treated with sub-MIC levofloxacin, rifampicin, or their combination (Figure 11, Table S1). These EPNs showed treatment-specific expression patterns, indicating distinct adaptive responses of biofilm-associated cells under antibiotic stress.

The EPN2 cluster comprised four proteins that consistently showed increased abundance across all treatment conditions: formate-tetrahydrofolate ligase (Fhs), general stress protein (YdaG), peroxide-responsive repressor (PerR), and halothane dehalogenase-like hydrolase (YcsE). These proteins appear to be part of a core stress response associated with sub-MIC antibiotic exposure. Fhs plays a central role in folate-mediated one-carbon metabolism and is essential for purine biosynthesis, methionine and serine production, and Met-tRNA formation [36]. YdaG, a part of the SigB regulon, is a general stress protein involved in resistance to acid, heat, and oxidative stress [37]. Increased YdaG expression suggests a role in the cellular response to antibiotic-induced stress. PerR, a member of the iron uptake regulator family, regulates genes linked to peroxide detoxification and metal ion homeostasis [38], suggesting that oxidative stress may be a common outcome of sub-MIC antibiotic exposure. YcsE, a 5’-nucleotidase within the haloacid halogenase superfamily [39], is associated with nucleotide metabolism and can promote nucleoside recycling under conditions of nucleotide imbalance. The consistently increased abundance of these four proteins suggests a shared protective response in *S. epidermidis* biofilms under sublethal antibiotic pressure and highlights their potential relevance as targets for biofilm control strategies.

The EPN5 cluster (72 proteins) was selectively overexpressed in response to rifampicin mono-treatment. Functional enrichment revealed significant activation of proteins involved in nucleotide transport and metabolism [F] and amino acid transport and metabolism [E], consistent with rifampicin-induced metabolic activation. The EPN9 cluster (66 proteins), specific to combination treatment, was enriched for proteins linked to amino acid metabolism [E] and energy production and conversion [C], indicating that dual-antibiotic exposure may increase metabolic activity. The EPN4 (68 proteins), which showed increased protein abundance in both rifampicin alone and the combination treatments, was enriched in translation, ribosome structure and biogenesis [J], and nucleotide metabolism [F]. This expression pattern is consistent with enhanced activity of protein synthesis and nucleotide processing pathways following rifampicin exposure. The EPN3 (23 proteins) showed uniformly decreased protein abundance in all treatment groups. Functional classification analysis results showed notable suppression of proteins associated with carbohydrate metabolism [G] and nucleotide transport/metabolism [F], suggesting a shared metabolic vulnerability associated with sub-MIC antibiotic exposure. The EPN7 (107 proteins), which showed markedly decreased protein abundance in levofloxacin mono-treatment, included proteins involved in nucleotide metabolism [F], energy production [C], and transcription [K], consistent with the broad suppressive effects of levofloxacin on metabolic and genetic information processing pathways.

### 3.6. Antimicrobial Resistance-Associated Proteins

Proteomic analysis showed changes in the expression of antimicrobial resistance-associated proteins in *S. epidermidis* biofilms following sub-MIC antibiotic treatment. Two major efflux pump proteins, NorA and NorC, consistently showed markedly decreased abundance across all treatment conditions (fold change: −13.29; Table 1). These transporters play a key role in multidrug resistance by actively pumping antibiotics out of the cell [40]. Reduced expression of these transporters may increase susceptibility by limiting efflux-mediated resistance.

Conversely, several antimicrobial resistance-associated proteins showed considerably increased abundance in rifampicin mono-treatment and combination treatment groups. These included the response regulator (WalR), penicillin-binding protein (MecA), dihydrofolate reductases (FolA-1 and FolA-2), putative drug transporter (SdrM), enzymes involved in peptidoglycan and fatty acid biosynthesis (MurAA and FabI), and ABC transporter (BceA), all of which showed a fold change of 9.97. This coordinated increase in protein abundance is consistent with an adaptive response that supports cell wall integrity, metabolic flexibility, and response to rifampicin-induced stress.

Additional antimicrobial resistance-associated proteins showed treatment-specific regulation. Following levofloxacin mono-treatment, the global transcriptional regulator (MgrA), which controls virulence and antimicrobial resistance pathways [41], showed notably decreased protein abundance (−13.29-fold), suggesting suppression of key regulatory networks under fluoroquinolone stress. In the combination treatment group, D-alanine-D-alanine ligase (Ddl) expression was markedly increased (9.97-fold), suggesting a compensatory response to maintain cell wall synthesis under dual antibiotic pressure. These findings show that sub-MIC antibiotic exposure produces both compensatory and inhibitory changes in antimicrobial resistance-associated proteins.

### 3.7. Virulence-Associated Proteins

In addition to antimicrobial resistance-associated proteins, several virulence-related proteins were differentially expressed following sub-MIC antibiotic exposure. Rifampicin, both alone and in combination with levofloxacin, considerably increased the expression of proteins involved in metabolic adaptation and stress response pathways. In particular, the expression of proteins linked to purine biosynthesis (PurM, PurL), urease-mediated nitrogen metabolism (UreC, UreG), and peptidoglycan remodeling (Mpl) showed significantly increased abundance (9.97-fold; Table 2). These pathways support anabolic processes that are important for bacterial persistence in nutrient-limited or stressful environments, such as biofilms.

In contrast, levofloxacin mono-treatment led to a marked decrease in the expression of several key metabolic enzymes, each exhibiting a fold change of –13.29. These included 6-phosphogluconate dehydrogenase (Gnd), aldehyde-alcohol dehydrogenase (Adh), catabolite regulatory protein A (CcpA), acid sugar phosphatase (NagD), and aconitate hydratase (CitB).

Two virulence-associated biosynthetic enzymes—carbamoyl phosphate synthase small chain (CarA) and aspartate-semialdehyde dehydrogenase (Asd)—uniquely showed increased abundance in the combination treatment group, with both enzymes showing a 9.97-fold increase in abundance. These enzymes catalyze key steps in pyrimidine nucleotide and amino acid biosynthesis, respectively, and their induction is consistent with a compensatory metabolic response to dual antibiotic stress. In addition, recombinase A (RecA), a key component of the bacterial SOS response, DNA repair, and horizontal gene transfer mechanisms [42], showed markedly decreased protein abundance (−13.29) in both the levofloxacin mono-treatment and combination treatment groups. This reduction in RecA expression suggests that sub-MIC exposure may limit genomic plasticity and adaptive responses under antibiotic stress.

### 3.8. Integration of Phenotypic and Proteomic Responses to Sub-MIC Antibiotic Exposure

Integrating phenotypic and proteomic analyses provided a more comprehensive understanding of how sub-MIC antibiotic exposure influences *S. epidermidis* biofilm physiology than either approach alone. The significant reduction in viable biofilm cells observed following rifampicin treatment was accompanied by increased expression of proteins involved in translation, energy production, and amino acid and nucleotide biosynthesis. This response likely reflects an adaptive effort by surviving cells to maintain essential cellular functions under rifampicin-induced transcriptional stress. Thus, the proteomic findings not only support the observed decrease in CFU counts but also offer mechanistic insight into the antibiofilm activity of rifampicin at subinhibitory concentrations. Although biofilm cells activated multiple metabolic and biosynthetic pathways in response to antibiotic stress, these compensatory responses were insufficient to fully overcome the inhibitory effects of RNA polymerase disruption, resulting in a measurable loss of biofilm viability.

The relationship between phenotypic and proteomic responses was less straightforward for levofloxacin mono-treatment. Despite the absence of a significant reduction in either viable cell counts or total biofilm biomass, levofloxacin triggered widespread decreases in the abundance of proteins involved in central metabolism, stress response, and protein synthesis. This difference can, however, be explained physiologically. CFU-based assays measure viability but cannot capture the functional state of surviving cells. The broad suppression of metabolic and stress-response proteins observed following levofloxacin treatment is more indicative of a transition toward a dormant or phenotypically tolerant state than of active cellular growth. Although this physiological shift is not apparent from CFU measurements, it is clearly reflected in the proteomic data. Notably, this pattern of antibiotic-induced quiescence has been documented in other biofilm-forming staphylococci and may reflect a strategy used by biofilm-forming staphylococci to tolerate antibiotic exposure [43,44].

Combination treatment significantly reduced both viable cell counts and biofilm biomass, and its proteomic profile closely resembled that of rifampicin alone. The persistence of a rifampicin-like expression pattern suggests that rifampicin is the principal driver of the antibacterial response, whereas levofloxacin may contribute to biofilm disruption through mechanisms that are not fully reflected in the proteomic data. Taken together, the phenotypic and proteomic results offer complementary insight into how *S. epidermidis* biofilms respond to sub-MIC antibiotic exposure. However, future studies using biological replicates will be needed to statistically confirm the treatment-related proteomic patterns observed here.

## 4. Conclusions

Levofloxacin and rifampicin affected *S. epidermidis* biofilm physiology through different mechanisms at 1/8× MIC, and their combination produced stronger antibiofilm effects than either agent alone. Notably, levofloxacin showed little effect on biomass or viable cell counts despite causing widespread suppression of proteins involved in metabolism, translation, and stress response. This pattern is more indicative of a dormant, antibiotic-tolerant state than of active bacterial killing and would not be apparent from CFU-based measurements alone. In contrast, rifampicin stimulated broad metabolic and translational activity while simultaneously increasing the abundance of antimicrobial resistance-associated proteins, including MecA, WalR, and FolA. This finding suggests that subinhibitory rifampicin exposure can trigger adaptive cellular responses even when measurable antibiofilm activity is retained. Notably, the consistently increased abundance of Fhs, YdaG, PerR, and YcsE across all treatment conditions points to a conserved stress-response program activated by sublethal antibiotic exposure. Collectively, these findings demonstrate that the physiological impact of sub-MIC antibiotics is most accurately understood through the integration of phenotypic and proteomic analyses.

## Figures and Tables

**Figure 1:**
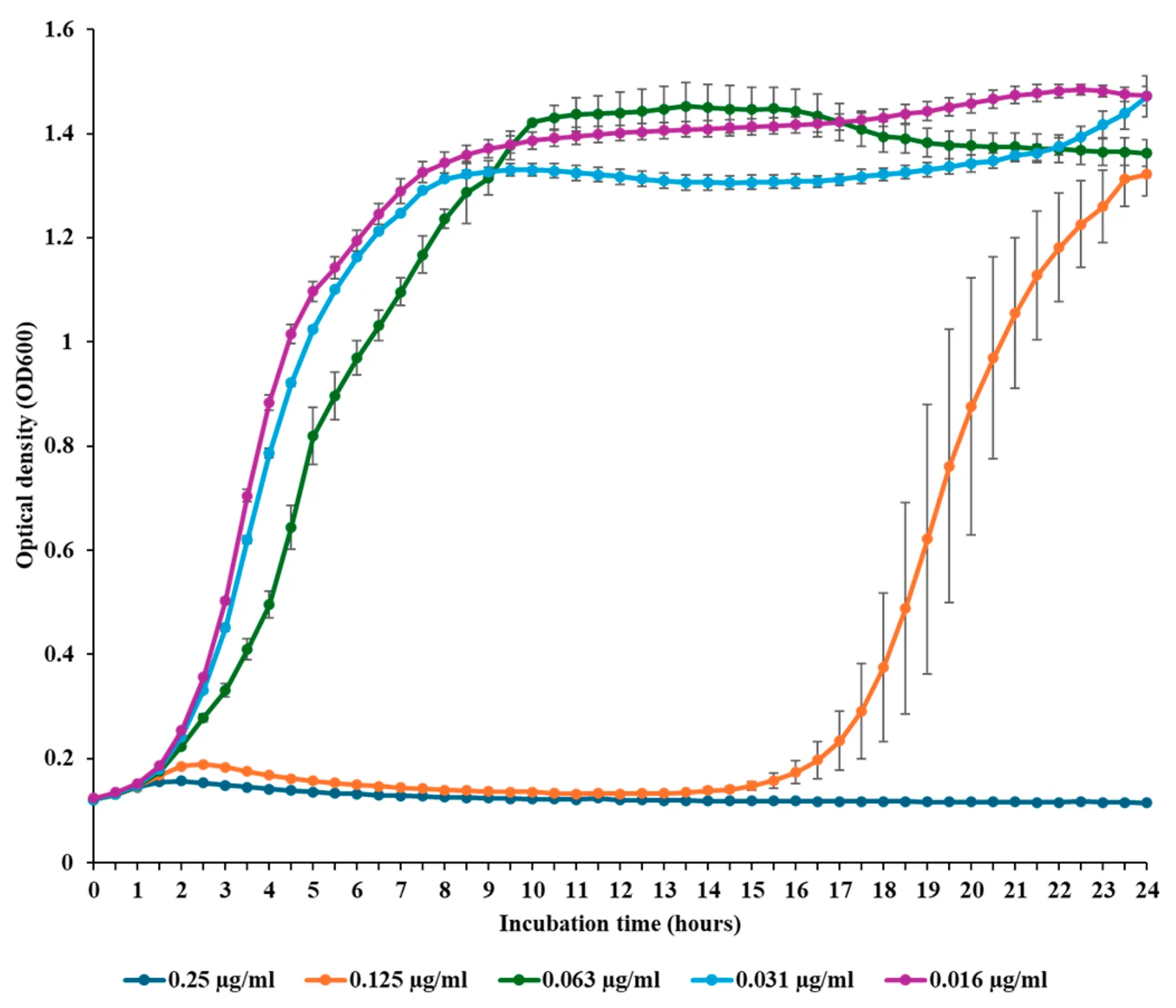
Determination of the minimum inhibitory concentration (MIC) of levofloxacin against *S. epidermidis* ATCC 35984. Data represent the mean ± SD of three independent measurements.

**Figure 2:**
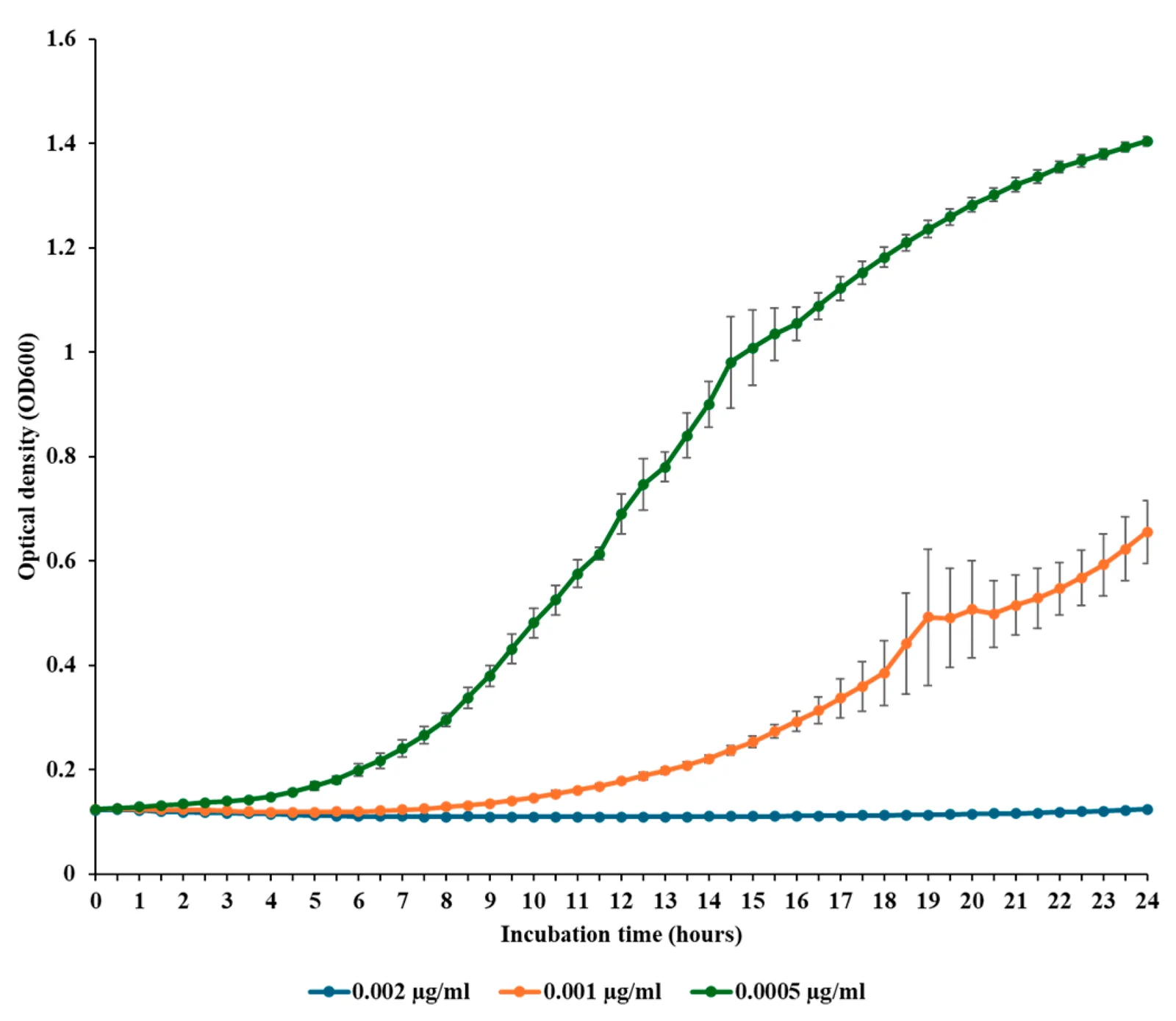
Determination of the minimum inhibitory concentration (MIC) of rifampicin against *S. epidermidis* ATCC 35984. Data represent the mean ± SD of three independent measurements.

**Figure 3:**
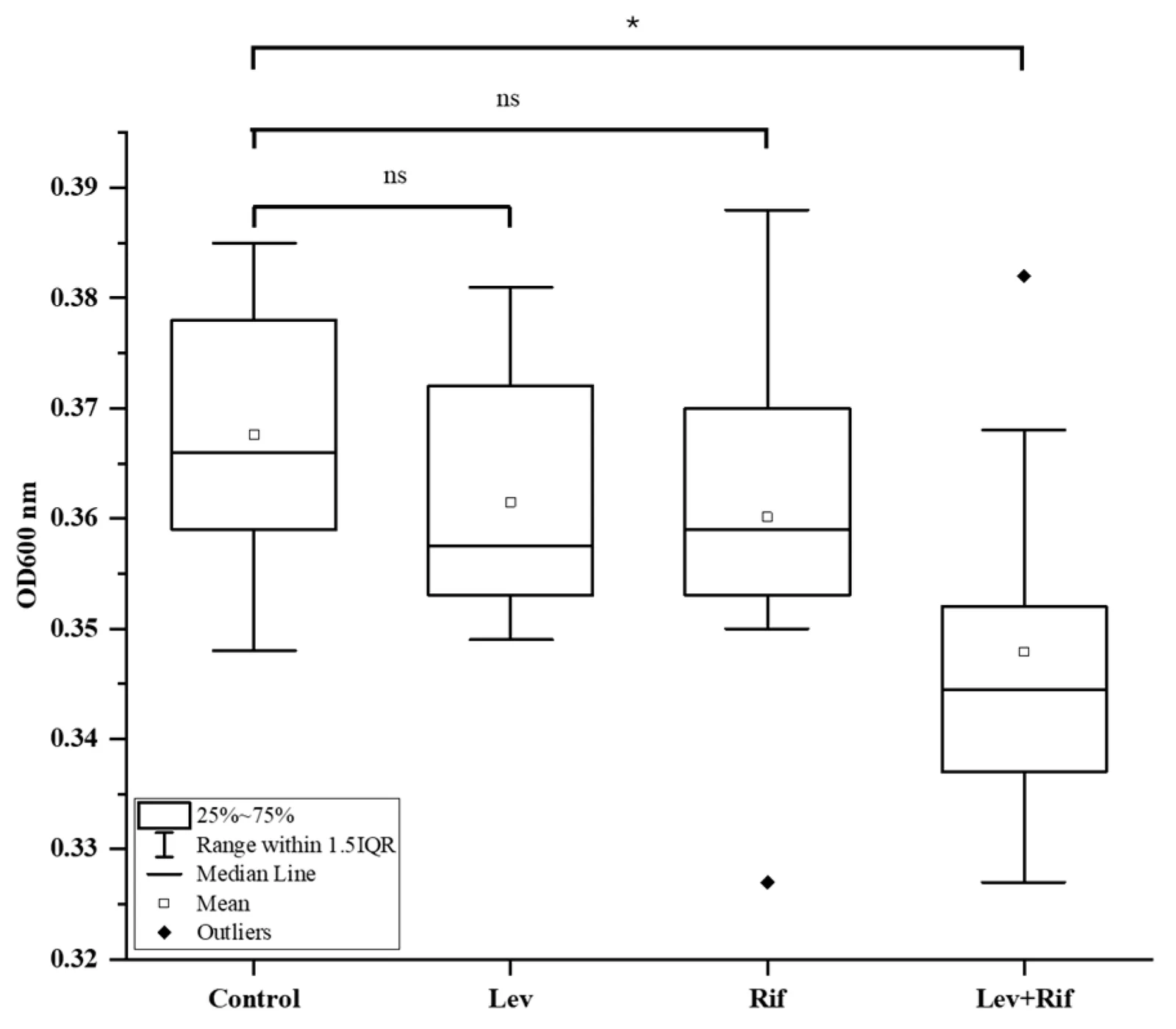
Biofilm biomass (OD600 nm) of *S. epidermidis* ATCC 35984 following treatment with sub-MIC levels of levofloxacin (**Lev**), rifampicin (**Rif**), or their combination (**Lev + Rif**). * *p* < 0.05; ns, not significant.

**Figure 4:**
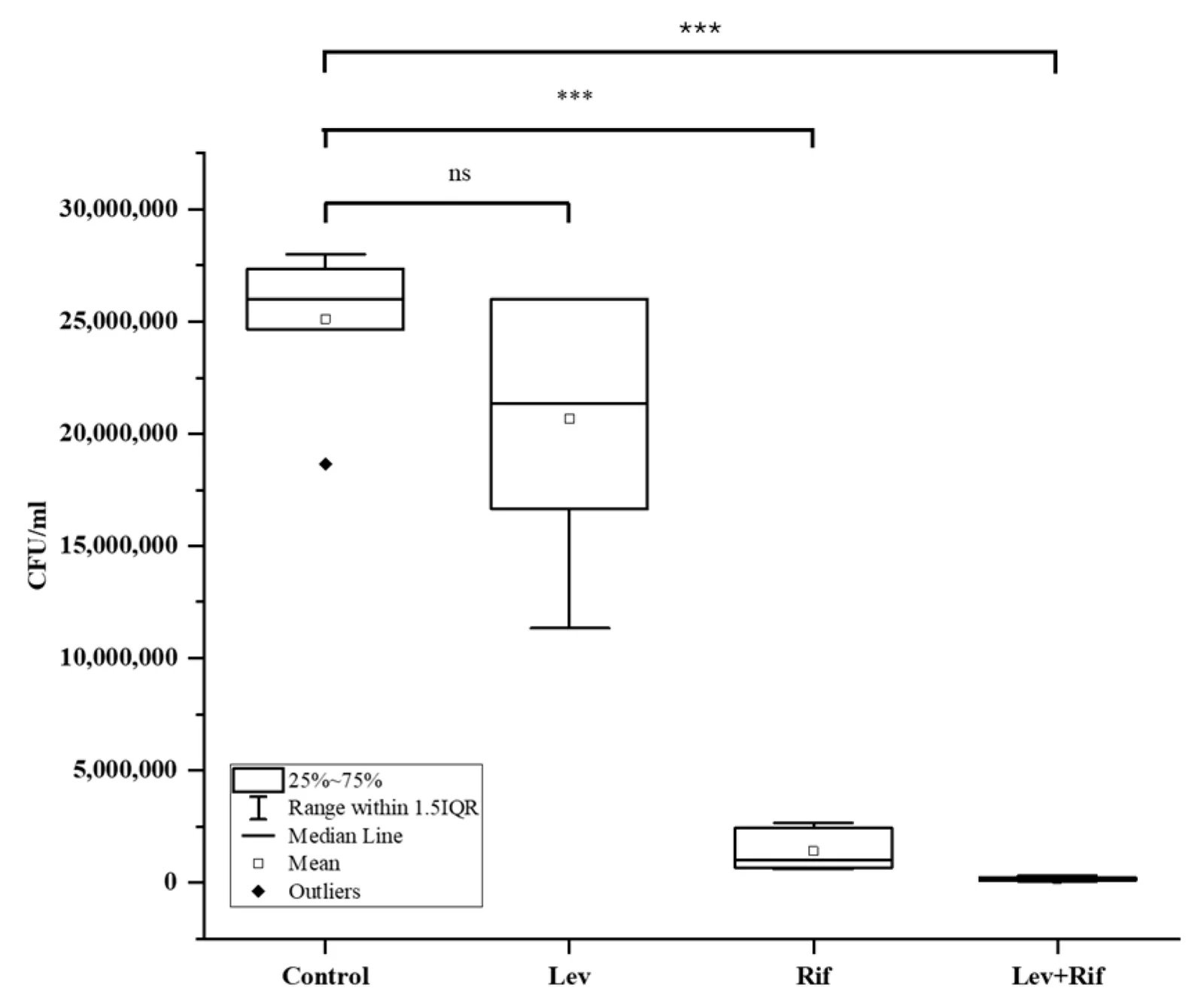
Colony forming unit (CFU) counts of *S. epidermidis* ATCC 35984 biofilms following treatment with sub-MIC levels of levofloxacin (**Lev**), rifampicin (**Rif**), or their combination (**Lev + Rif**). *** *p* < 0.001; ns, not significant.

**Figure 5:**
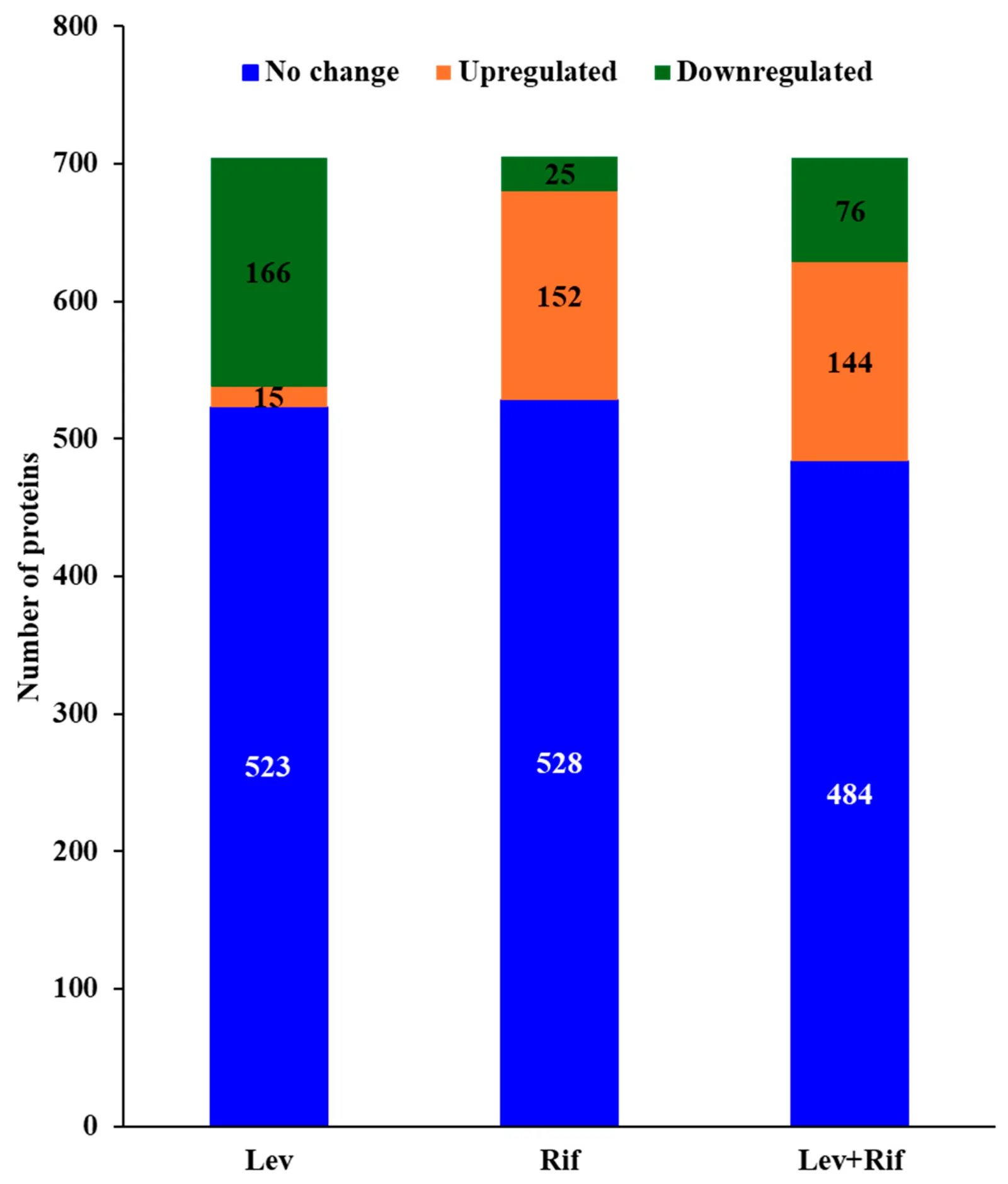
Distribution of differentially expressed proteins in *S. epidermidis* ATCC 35984 biofilms following treatment with sub-MIC levels of levofloxacin (**Lev**), rifampicin (**Rif**), or their combination (**Lev + Rif**).

**Figure 6:**
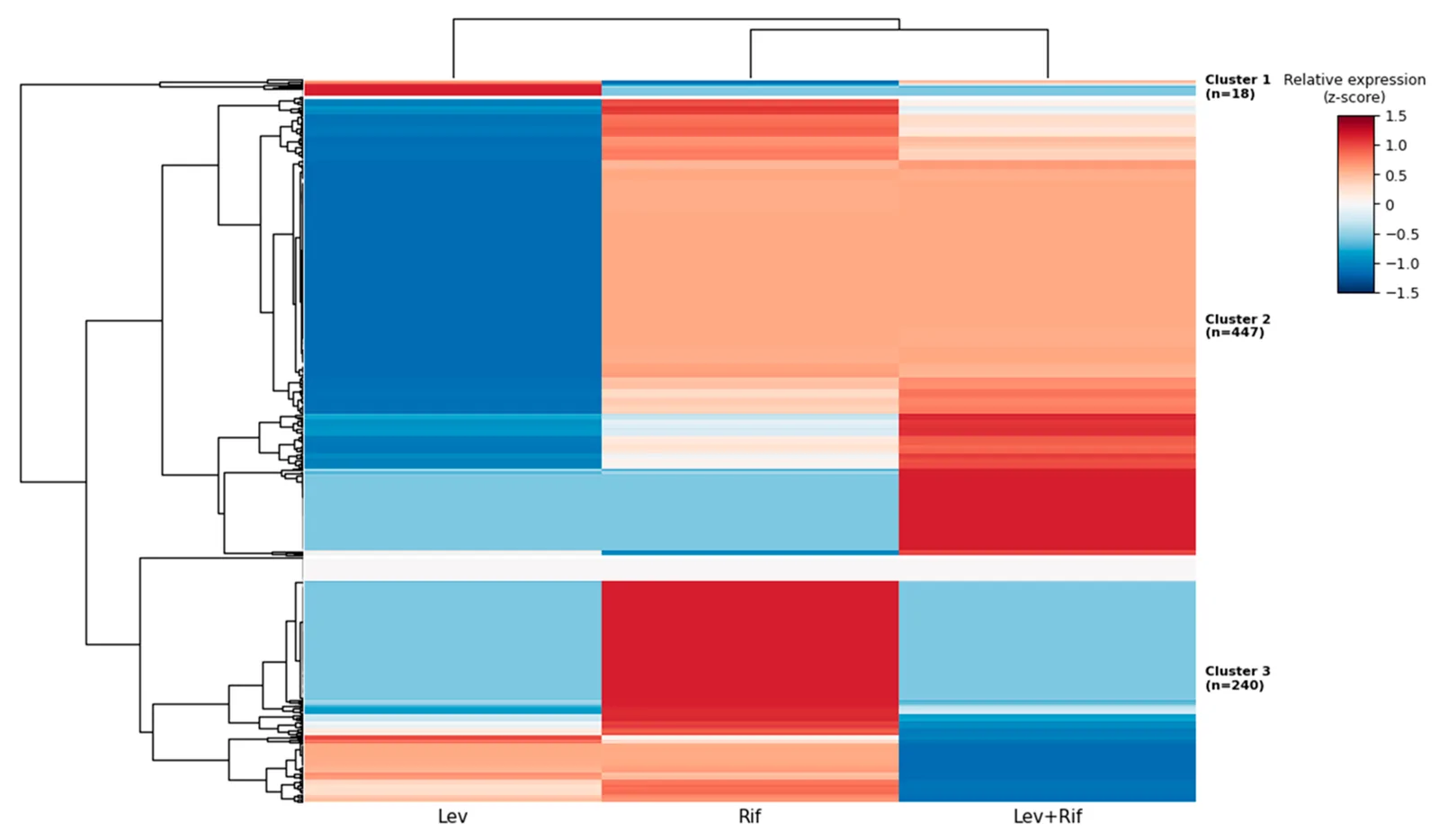
Hierarchical clustering and expression profiles of *S. epidermidis* ATCC 35984 biofilm proteins. The heatmap illustrates proteomic shifts following exposure to sub-MIC levels of levofloxacin (**Lev**), rifampicin (**Rif**), or their combination (**Lev + Rif**). The three major protein clusters (Clusters 1–3) are indicated in the figure. The color scale represents relative protein abundance, with red denoting increased abundance and blue denoting decreased abundance relative to the mean abundance of each protein.

**Figure 7:**
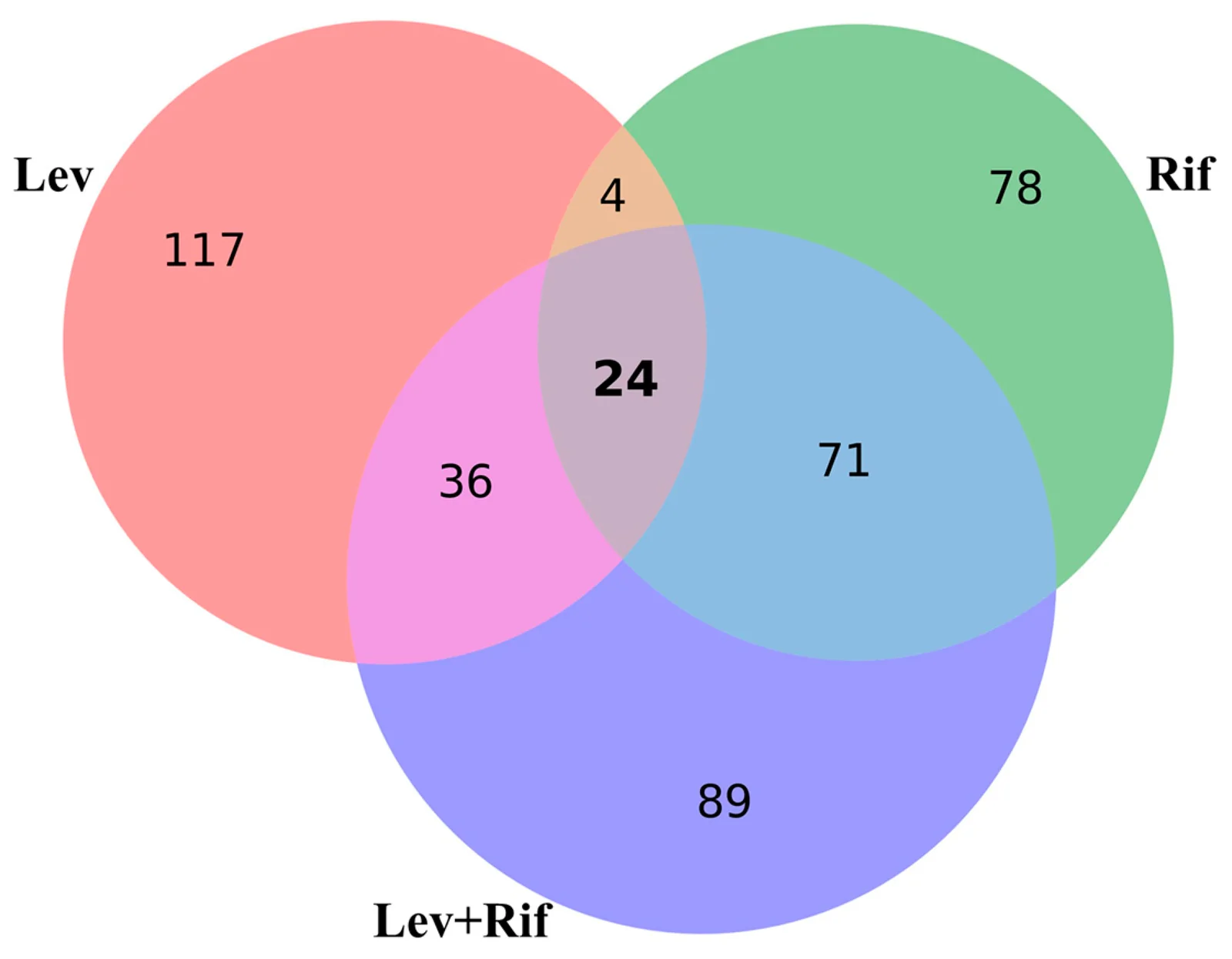
Venn diagram of differentially expressed proteins in *S. epidermidis* ATCC 35984 biofilms following treatment with sub-MIC levels of levofloxacin (**Lev**), rifampicin (**Rif**), or their combination (**Lev + Rif**). Numbers in parentheses indicate the total number of differentially expressed proteins identified at each time point.

**Figure 8:**
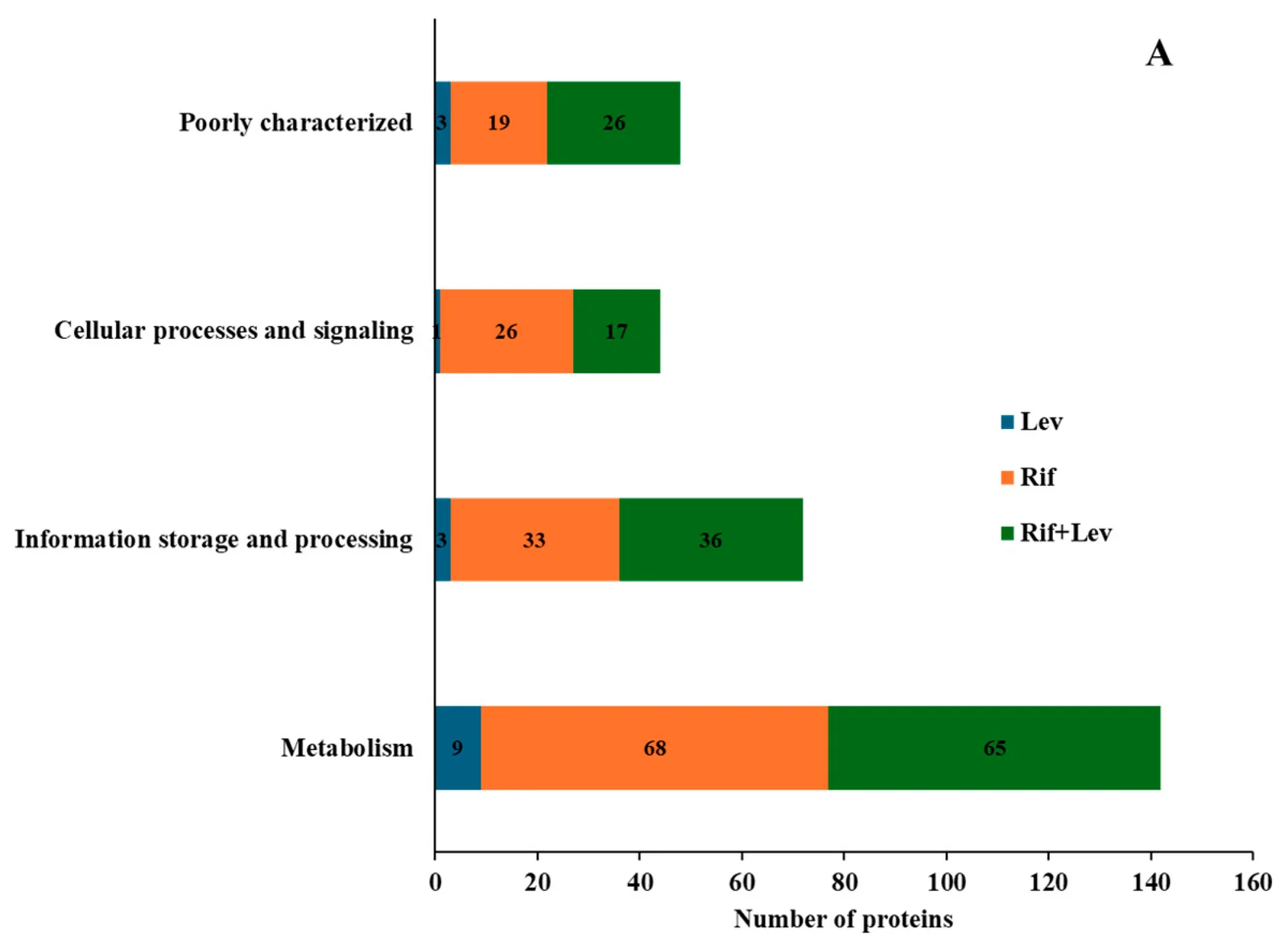

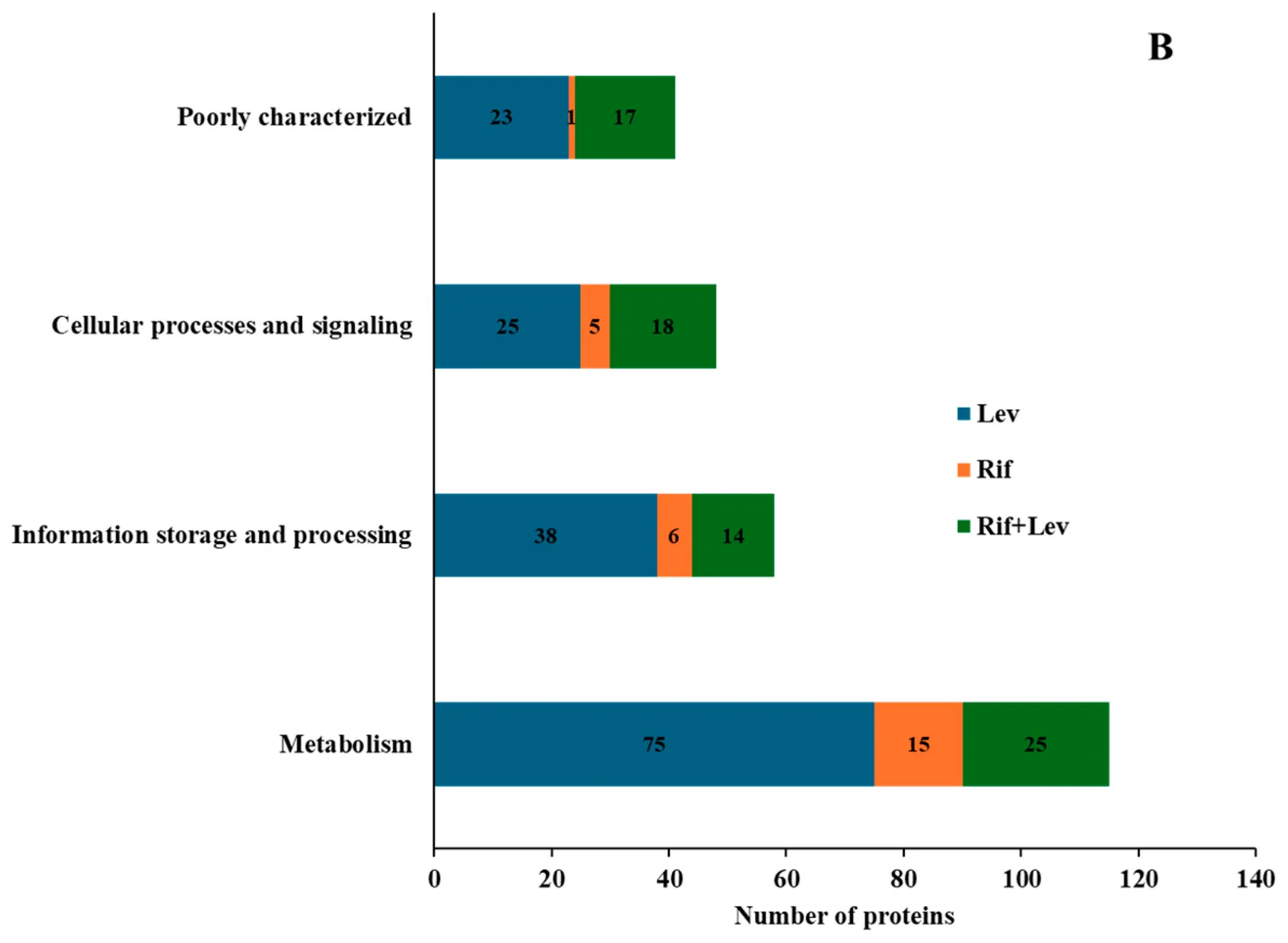
Major COG functional classification of differentially expressed proteins in *S. epidermidis* ATCC 35984 biofilms following treatment with sub-MIC levels of levofloxacin (**Lev**), rifampicin (**Rif**), or their combination (**Lev + Rif**). (**A**): Proteins showing increased abundance, (**B**): Proteins showing decreased abundance.

**Figure 9:**
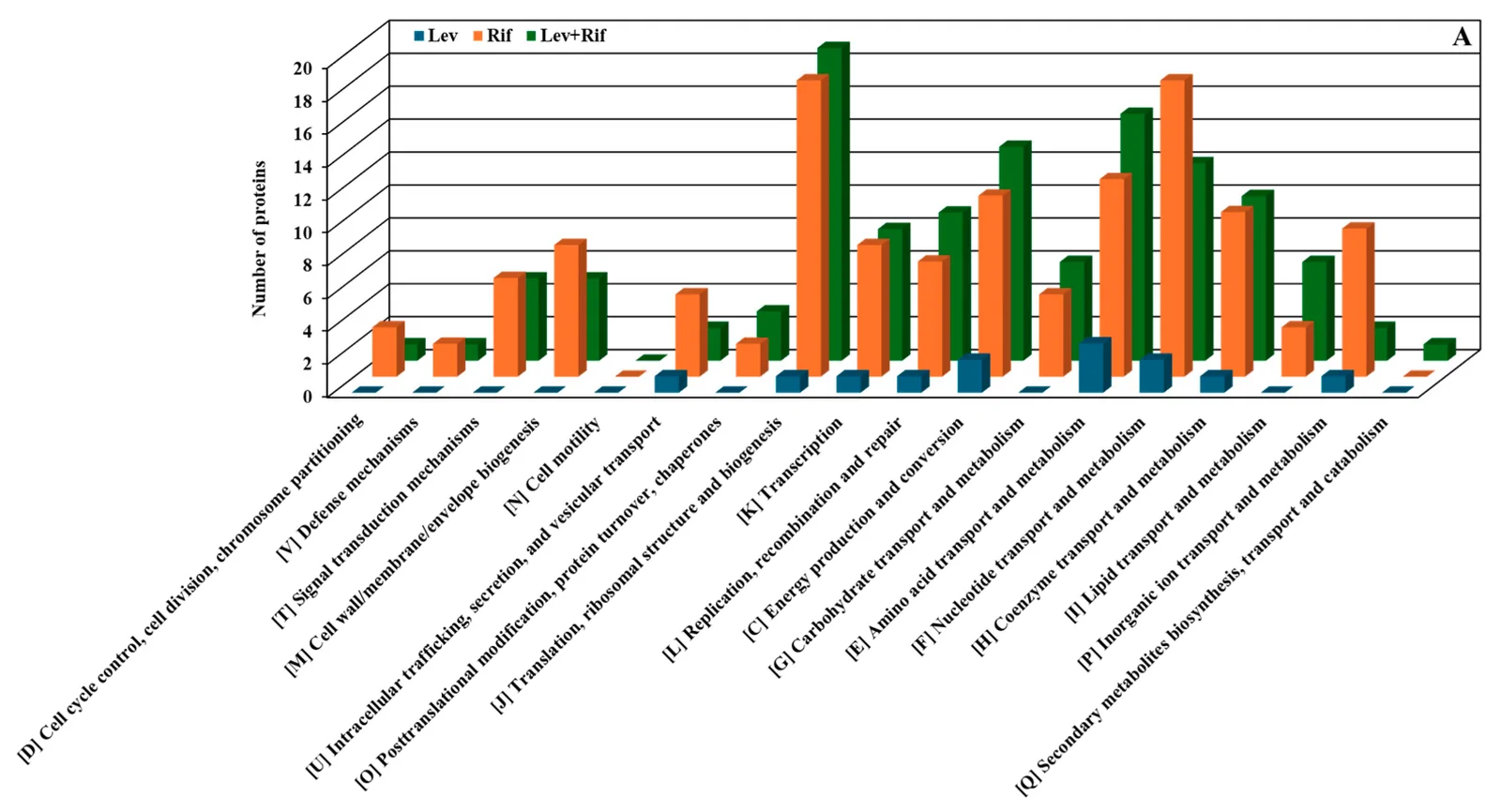

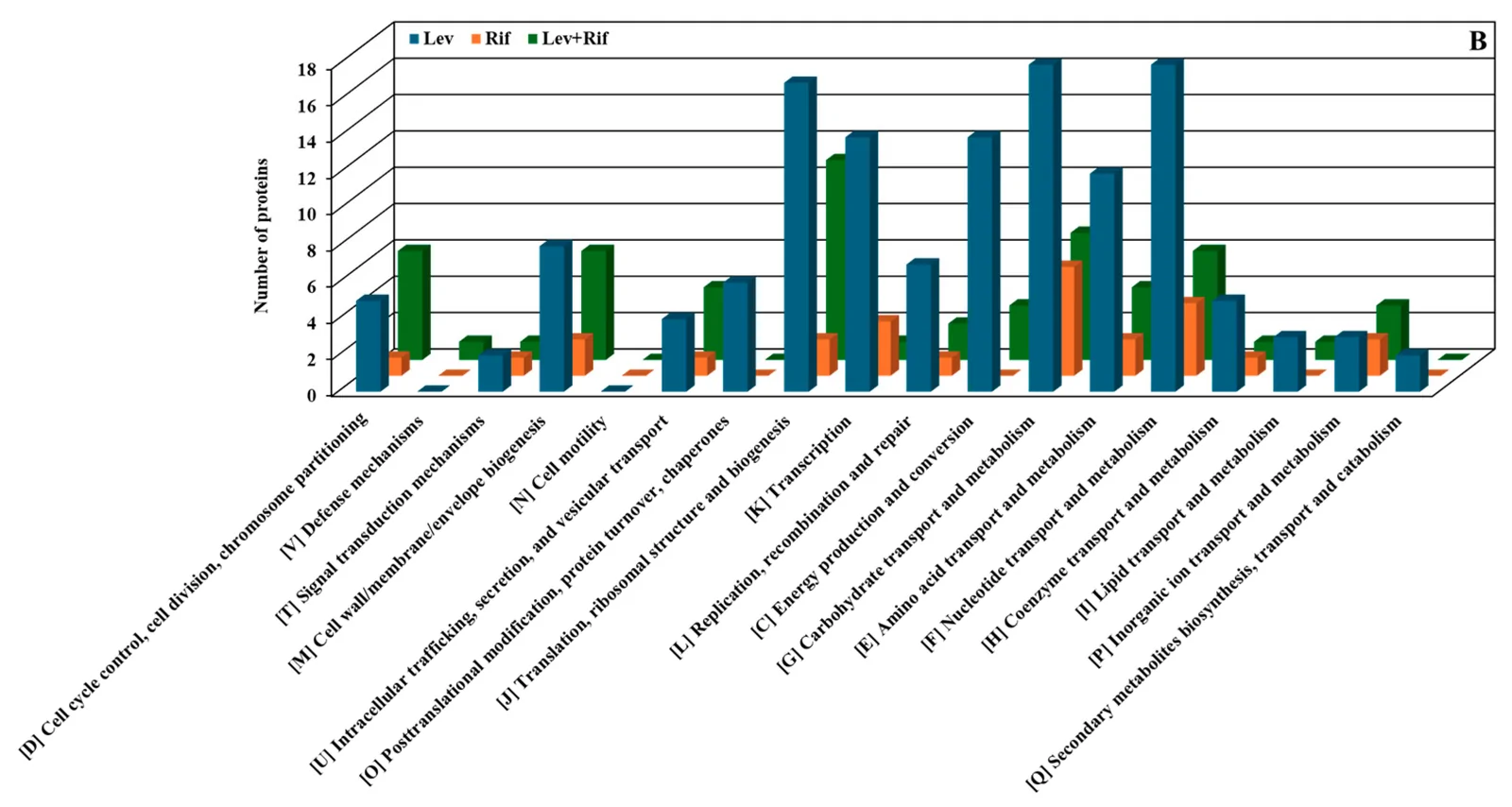
Detailed COG functional classification of differentially expressed proteins in *S. epidermidis* ATCC 35984 biofilms following treatment with sub-MIC levels of levofloxacin (**Lev**), rifampicin (**Rif**), or their combination (**Lev + Rif**). (**A**): Proteins showing increased abundance, (**B**): Proteins showing decreased abundance. The poorly characterized category (S) was excluded.

**Figure 10:**
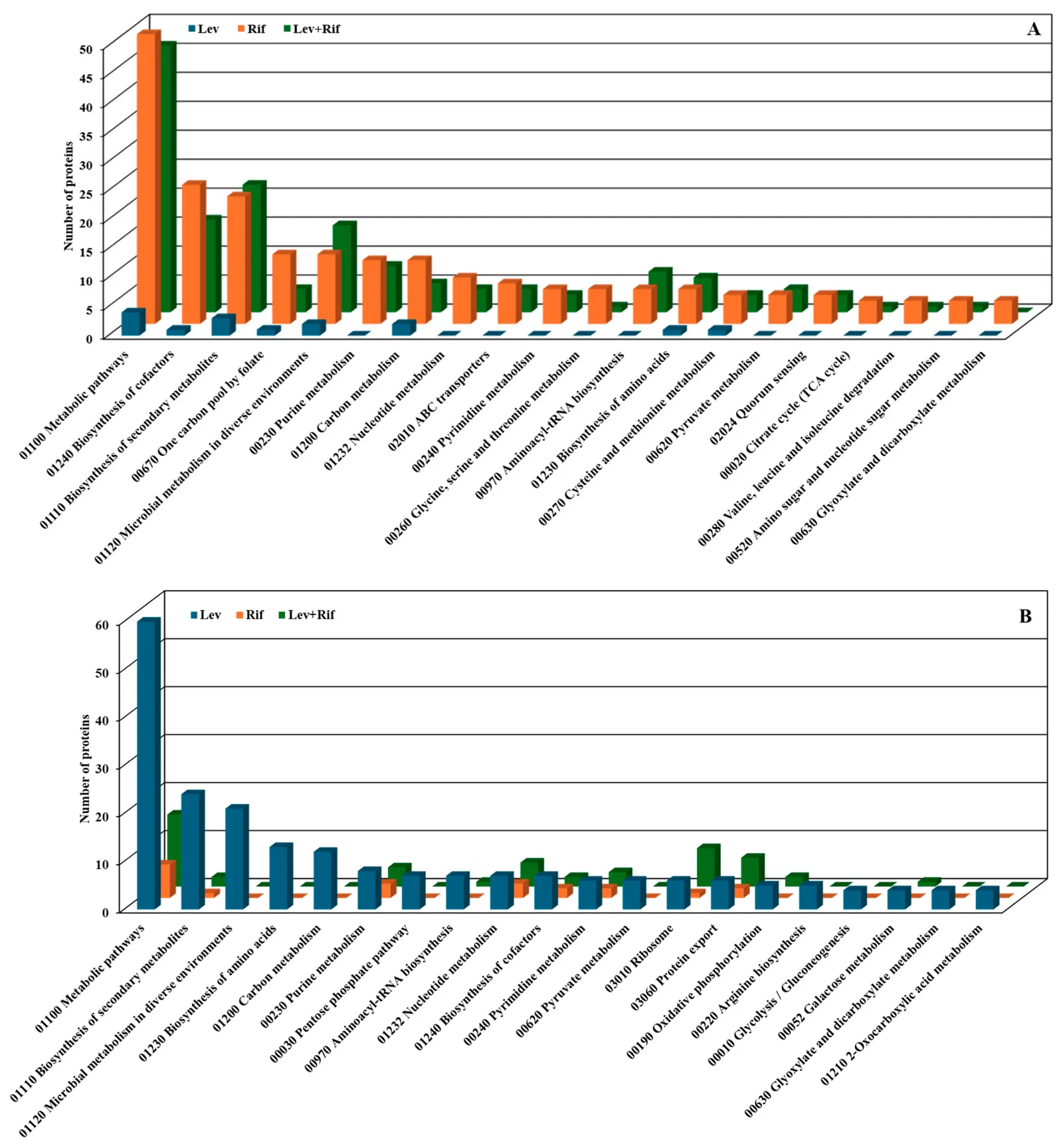
Top 20 KEGG pathways associated with differentially expressed proteins in *S. epidermidis* ATCC 35984 biofilms following treatment with sub-MIC levels of levofloxacin (**Lev**), rifampicin (**Rif**), or their combination (**Lev + Rif**). (**A**): Proteins showing increased abundance, (**B**): Proteins showing decreased abundance.

**Figure 11:**
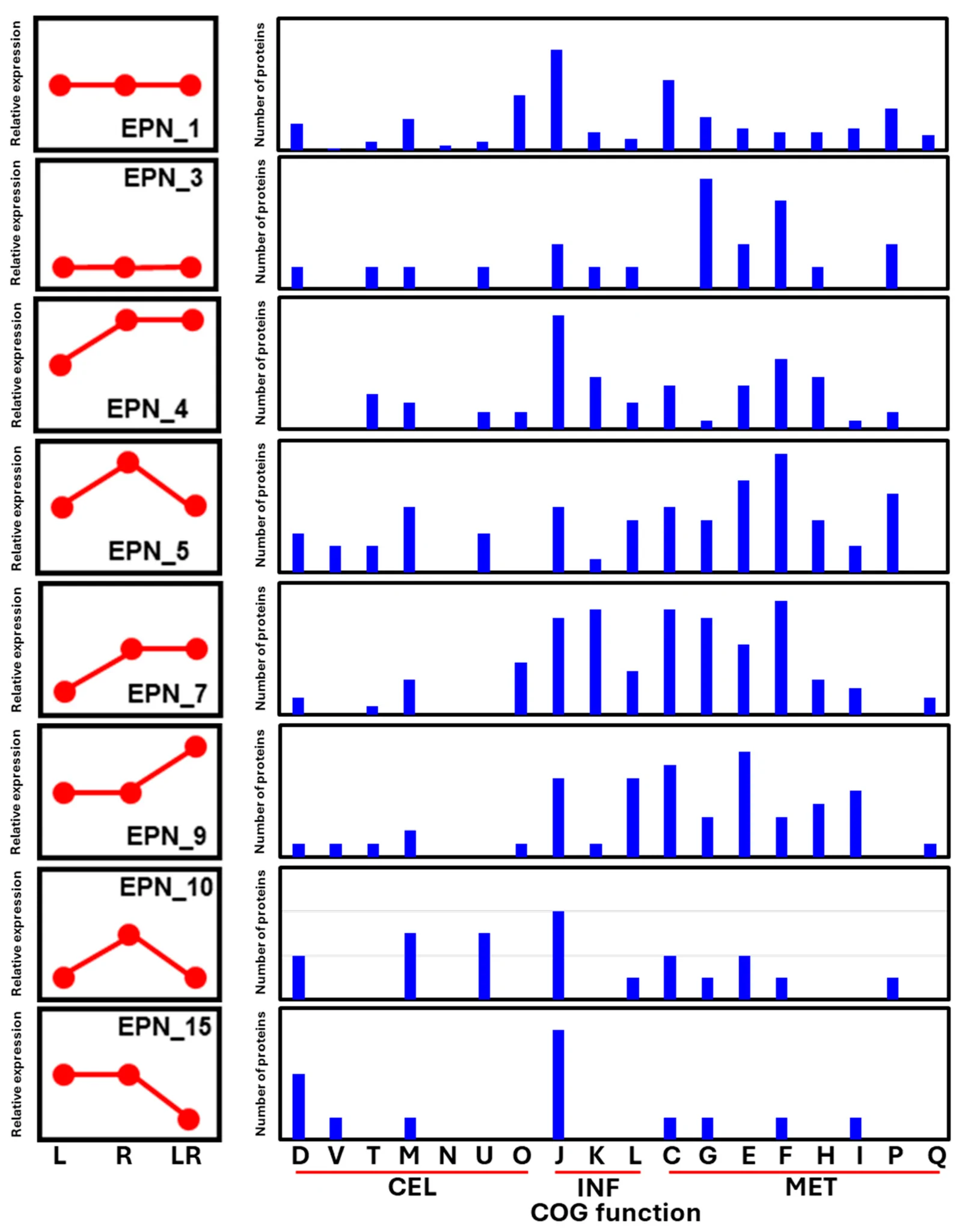
Expression pattern (EPN) clusters and associated COG functional profiles of proteins in *S. epidermidis* ATCC 35984 biofilms following treatment with sub-MIC levels of levofloxacin (**Lev**), rifampicin (**Rif**), or their combination (**Lev + Rif**). For each EPN cluster, the left panel shows the relative expression pattern across the three treatment conditions (L, levofloxacin; R, rifampicin; LR, levofloxacin + rifampicin). The *y*-axis represents relative protein expression compared with the untreated control, with upward and downward shifts indicating upregulation and downregulation, respectively, and the midline representing no change. The right panel shows the number of proteins assigned to each COG functional category (*y*-axis: Number of proteins). The two panels use independent y-axes because they represent different measurements. Abbreviations: **L**, levofloxacin; **R**, rifampicin; **LR**, levofloxacin + rifampicin. Functional groupings: **CEL**, cellular processes and signaling; **INF**, information storage and processing; **MET**, metabolism. COG categories: **D**, cell cycle control, cell division, chromosome partitioning; **V**, defense mechanisms; **T**, signal transduction mechanisms; **M**, cell wall/membrane/envelope biogenesis; **N**, cell motility; **U**, intracellular trafficking, secretion, and vesicular transport; **O**, posttranslational modification, protein turnover, chaperones; **J**, translation, ribosomal structure, and biogenesis; **K**, transcription; **L**, replication, recombination, and repair; **C**, energy production and conversion; **G**, carbohydrate transport and metabolism; **E**, amino acid transport and metabolism; **F**, nucleotide transport and metabolism; **H**, coenzyme transport and metabolism; **I**, lipid transport and metabolism; **P**, inorganic ion transport and metabolism; **Q**, secondary metabolite biosynthesis, transport, and catabolism. The poorly characterized category (**S**) was excluded.

**Table 1:** Differentially expressed proteins associated with antimicrobial resistance in *S. epidermidis* ATCC 35984 biofilms following treatment with sub-MIC levels of levofloxacin, rifampicin, or their combination.

Protein ID	Protein Description	Gene Name	COG	KEGG	Fold-Ratio		
					Lev	Rif	Lev + Rif
Q5HLG4	Quinolone resistance protein	*norC*	EGP	ko:K08170	−13.29	−13.29	−13.29
Q5HR40	Quinolone resistance protein	*norA*	EGP	ko:K19576	−13.29	−13.29	−13.29
Q5HK18	Transcriptional regulatory protein	*walR*	K	ko:K07668	ND	9.97	9.97
Q5HK31	Penicillin-binding protein 2	*mecA*	M	ko:K02545	ND	9.97	9.97
Q5HPB1	Dihydrofolate reductase	*folA-1*	H	ko:K00287	ND	9.97	ND
Q5HMP7	Dihydrofolate reductase	*folA-2*	H	ko:K00287	ND	9.97	ND
Q5HM61	Drug transporter, putative	*sdrM*	EGP	ko:K18935	ND	9.97	ND
Q5HMC2	UDP-N-acetylglucosamine 1-carboxyvinyltransferase 1	*murAA*	M	ko:K00790	ND	9.97	ND
Q5HQF2	Enoyl-[acyl-carrier-protein] reductase [NADPH]	*fabI*	I	ko:K00208	ND	9.97	ND
Q5HR79	ABC transporter, ATP-binding protein	*bceA*	V	ko:K02003	ND	9.97	ND
Q5HRL0	DNA-directed RNA polymerase subunit beta	*rpoB*	K	ko:K03043	−13.29	0.13	0.17
Q5HR51	HTH-type transcriptional regulator MgrA	*mgrA*	K	ko:K18906	−13.29	0.01	0.08
Q5HRK9	DNA-directed RNA polymerase subunit beta	*rpoC*	K	ko:K03046	−13.29	−0.28	−0.48
Q5HMD8	D-alanine--D-alanine ligase	*ddl*	F	ko:K01921	ND	ND	9.97
Q5HLN7	Membrane-associated protein	*tcaA*	S	ko:K21463	−13.29	0.05	−13.29
Q5HRK7	Small ribosomal subunit protein uS12	*rpsL*	J	ko:K02950	−0.21	0.00	−13.29

Lev: levofloxacin; Rif: rifampicin; Lev + Rif: combination of levofloxacin and rifampicin. ND indicates that the protein was not detected in either the control or treated samples.

**Table 2:** Differentially expressed proteins associated with virulence in *S. epidermidis* ATCC 35984 biofilms following treatment with sub-MIC levels of levofloxacin, rifampicin, or their combination.

Protein ID	Protein Description	Gene Name	COG	KEGG	Fold-Ratio		
					Lev	Rif	Lev + Rif
Q5HQ99	Phosphoribosylformylglycinamidine cyclo-ligase	*purM*	F	ko:K01933	ND	9.97	9.97
Q5HKU0	Neutral metalloproteinase	*mpl*	E	ko:K01400	ND	9.97	9.97
Q5HLV8	Urease accessory protein	*ureG*	KO	ko:K03189	ND	9.97	9.97
Q5HKP8	Poly-beta-1,6-N-acetyl-D-glucosamine N-deacetylase	*icaB*	G	ko:K21478	ND	9.97	ND
Q5HQA1	phosphoribosylformylglycinamidine synthase II	*purL*	F	ko:K01952	ND	9.97	ND
Q5HQ23	dITP/XTP pyrophosphatase	*rdgB*	F	ko:K02428	−13.29	0.02	0.11
Q5HP42	6-Phosphogluconate dehydrogenase, decarboxylating	*gnd*	G	ko:K00033	−13.29	0.20	0.15
Q5HR06	Aldehyde-alcohol dehydrogenase	*adh*	C	ko:K04072	−13.29	0.06	0.05
Q5HNH3	Catabolite control protein A	*ccpA*	K	ko:K02529	−13.29	0.02	0.08
Q5HQN3	Acid sugar phosphatase	*nagD*	G	ko:K01101	−13.29	0.31	0.47
Q5HPJ0	Aconitate hydratase	*citB*	C	ko:K01681	−13.29	0.10	0.14
Q5HR51	MarR family transcriptional regulator	*mgrA*	K	ko:K18906	−13.29	0.01	0.08
Q5HPY9	Carbamoyl phosphate synthase small chain	*carA*	F	ko:K01956	ND	ND	9.97
Q5HLW1	Urease subunit alpha	*ureC*	E	ko:K01428	ND	ND	9.97
Q5HPE8	Aspartate-semialdehyde dehydrogenase	*asd*	E	ko:K00133	ND	ND	9.97
Q5HPQ6	Recombinase A	*recA*	L	ko:K03553	−13.29	0.12	−13.29

Lev: levofloxacin; Rif: rifampicin; Lev + Rif: combination of levofloxacin and rifampicin. ND indicates that the protein was not detected in either the control or treated samples.

## Data Availability

The original contributions presented in this study are included in the article/Supplementary Materials. Further inquiries can be directed to the corresponding author.

## Supplementary Materials

The following supporting information can be downloaded at: https://www.mdpi.com/article/10.3390/pathogens15101008/s1, Table S1: Total identified proteins; Table S2: KEGG pathways associated with proteins showing increased abundance; Table S3: KEGG pathways associated with proteins showing decreased abundance. Figure S1. Confocal laser scanning microscopy (CLSM) images of *S. epidermidis* ATCC 35984 biofilms following treatment with sub-MIC levels of levofloxacin (Lev), rifampicin (Rif), or their combination (Lev + Rif). The scale bar is 200 µm. A: untreated control, B: levofloxacin-treated, C: rifampicin-treated, D: levofloxacin + rifampicin combination-treated.
