# Supplementary material for: Proteomic Insights into Antibiotic Resistance and Biofilm Adaptation of Staphylococcus epidermidis to Subinhibitory Levofloxacin, Rifampicin, and Combination Treatment
Source: Pathogens. 2026 Sep 24;15(10):1008. doi: 10.3390/pathogens15101008 (PMC13708677; doi:10.3390/pathogens15101008)
Supplement: Supplementary file 1 [file pathogens-15-01008-s001.zip › Figure S1.pdf]

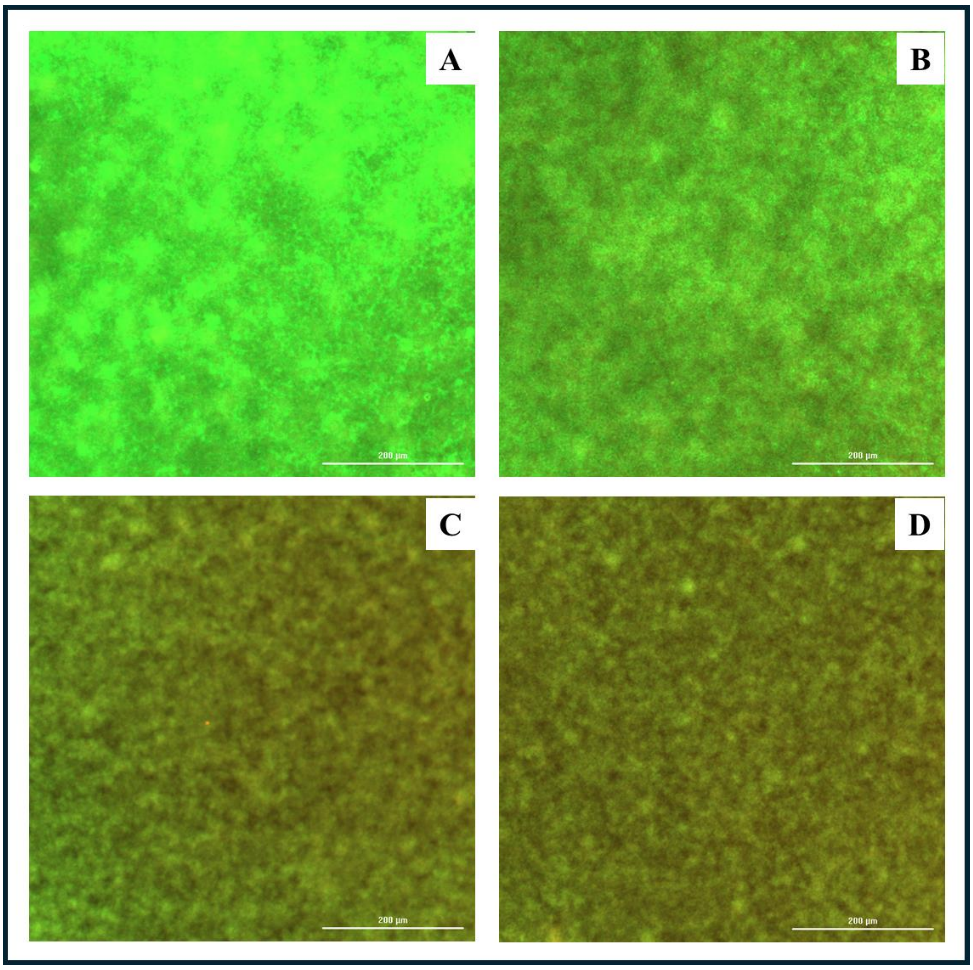

Figure S1. Confocal laser scanning microscopy (CLSM) images of S. epidermidis ATCC 35984 biofilms following treatment with sub-MIC levels of levofloxacin (**Lev**), rifampicin (**Rif**), or their combination (**Lev+Rif**). The scale bar is 200 µm. **A**: untreated control, **B**: levofloxacin-treated, **C**: rifampicin-treated, **D**: levofloxacin + rifampicin combination-treated.
